# Association between serum neurofilament light chain and periodontitis

**DOI:** 10.1007/s00784-024-05769-1

**Published:** 2024-06-12

**Authors:** Jing Zhao, Panwen Zhao

**Affiliations:** Department of Central Laboratory, Affiliated Hospital 6 of Nantong University, Yancheng Xindu Road 606#, Yancheng, Jiangsu Province PR China

**Keywords:** Serum neurofilament light, Serum albumin, Periodontitis, Biomarker, Mediation analysis

## Abstract

**Objectives:**

The association between serum neurofilament light chain (sNfL) and periodontitis remains unclear, and there is a need to examine the contribution of serum albumin (SA) in this association. The objective of the study is to investigate the correlation between sNfLand periodontitis, while examining the potential mediator role of SA in this association.

**Methods:**

The study, which included 1218 participants from the 2013–2014 National Health and Nutrition Examination Survey (NHANES), aimed to evaluate the association between sNfL and periodontitis through weighted multivariable logistic regression analysis, restricted cubic spline (RCS) models, and stratified models. In addition, mediation analysis was used to investigate the role of SA in mediating this association.

**Results:**

The multivariable logistic regression models revealed that sNfL was significantly linked to periodontitis (model 1: odds ratio [OR], 3.08, 95% confidence interval [CI], 1.48 to 6.39, model 2: OR, 3.69; 95% CI, 1.73 to 7.90, model 3: OR, 3.58, 95% CI, 1.52 to 8.43). The RCS models suggested a linear relationship between sNfL and periodontitis. The stratified analysis revealed no significant moderating effects (p-value > 0.05). The mediation analysis demonstrated that SA mediated the correlation between sNfL and periodontitis, with a mediation proportion of 10.62%.

**Conclusions:**

The results point to sNfL being a factor in the heightened risk of periodontitis. Additionally, SA may mediate the changes in periodontitis that are associated with sNfL.

**Clinical relevance:**

sNfL may contribute to the development of periodontitis by mediating changes in SA in humans.

## Introduction

Periodontitis is a chronic disorder that results from the accumulation of plaque biofilm on the teeth. This leads to an imbalance in the intraoral ecosystem, resulting in an abnormal host response that damages tissue and hinders effective bacterial clearance [1]. The success of periodontal treatment significantly relies on the elimination of supra- and subgingival biofilms from the teeth, as well as patient self-care practices to inhibit the reformation of biofilms [2]. Nonsurgical periodontal treatment, a primary intervention for individuals with periodontitis, when coupled with patient education and motivation in oral hygiene, results in a significant reduction in bleeding on probing, probing pocket depth, and clinical attachment loss [3]. It is well-documented that periodontitis is associated with several diseases, such as cardiovascular disease, rheumatoid arthritis, diabetes, and metabolic syndrome [4]. Other reports have suggested that despotic oral bacteria can release their products into the circulatory system, potentially crossing the blood-brain barrier and altering the gut microbiome. This can lead to inflammation and may even affect the brain through the gut-brain axis [5].

The neurofilament light chain (NfL) is a 68 kDa cylindrical protein that is a component of neurofilaments, which are responsible for preserving the structural stability of neurons. It is found in the cytoplasm of neurons and is released into the extracellular space when axonal damage occurs [6]. Recent studies have reported a significant association between alterations in serum neurofilament light (sNfL) levels and an increased risk of neurodegenerative diseases (NDs) [7–9]. Furthermore, emerging evidence suggests that NfL may serve as a biomarker in non-primary neurological diseases, including those related to perioperative care, human infectious diseases and intensive care units [10].

Albumin is a widely distributed globular protein found in vertebrates [11]. It is also a major component of extracellular fluids, such as lymph, interstitial, and cerebrospinal fluids [12]. Serum albumin (SA) is the most abundant protein in the blood, with a concentration of 30–50 mg/mL [13], indicating the overall health of an individual. As reported, SA provides protection to both neurons and glial cells while exhibiting antioxidant and anti-inflammatory activities [14]. Moreover, studies have indicated a significant relationship between SA and oral health [15–17]. The multifunctional features of SA remind us that it may play a mediating role in various disease processes.

Periodontitis, which affects 10–15% of adults, is an inflammatory condition that not only impacts oral health but also general health [18, 19]. Understanding its pathogenesis and contributing factors is crucial for effective prevention and management. Previous research has indicated the involvement of NfL in inflammation [20], suggesting a potential association between NfL and periodontitis. Additionally, albumin, a significant plasma protein with diverse physiological functions, has been linked to inflammatory responses [14, 21, 22]. Based on these connections, it is hypothesized that SA may play a mediating role in the relationship between NfL and periodontitis. To clarify the association between sNfL and periodontitis, while also examining the potential mediating effect of SA, we conducted an exploratory analysis based on data from the 2013–2014 National Health and Nutrition Examination Survey (NHANES). The objective of this research is to deepen our understanding of the pathogenesis of periodontitis, with the broader aim of offering theoretical insights for the treatment of this condition.

## Materials and methods

### Study population

The NHANES, an open database, was initiated by the National Center for Health Statistics (NCHS) in the United States to explore the relationship between nutritional status and the promotion of health and prevention of diseases. NHANES uses a stratified multistage probability sample, and all procedures have been approved by the NHANES Institutional Review Board. Furthermore, written informed consent was obtained from each participant. For more information about NHANES, visit the Centers for Disease Control and Prevention website at http://www.cdc.gov/nchs/nhanes/aboutnhanes.htm.

Our study used NHANES data from the 2013–2014 cycle (*n* = 10175) due to the accessibility of sNfL data only during that period. A total of 8104 individuals was excluded from the analysis due to missing sNfL data, while 676 individuals were excluded due to missing periodontitis data. Additionally, one individual was excluded for missing education level, 90 for missing family income to poverty ratio (PIR), 67 for missing drinking status, five for missing body mass index (BMI), and one for missing smoking status. After further exclusions of two cases for missing sleep duration, four for missing systemic immune-inflammation index (SII), and seven for pregnancy, the final sample consisted of 1218 participants.

### Assessment of sNfL concentration

Serum samples used for testing NfL concentrations were obtained from individuals aged 20 to 75 who participated in the 2013–2014 NHANES study, provided consent for their samples to be utilized in further research, and possessed surplus or pristine serum samples. Blood is collected from the participant’s arm veins by a trained phlebotomist. The volume of blood collected depends on the participant’s age. After collection, the blood is processed, divided into vials, and stored in the mobile examination center. Subsequently, the vials are refrigerated or frozen before being transported to various laboratories throughout the United States. The concentration of sNfL was assessed using a high-throughput acridinium ester (AE) immunoassay from Siemens Healthineers on the Atellica immunoassay system. The serum samples underwent incubation with AE-labeled antibodies that specifically target the NfL antigen, followed by the introduction of paramagnetic particles coated with capture antibodies. Subsequently, unbound AE-labeled antibodies were eliminated, and a chemiluminescence reaction was initiated using an acid and a base, resulting in quantifiable light emission.

### Assessment of periodontitis

Individuals aged 30 and over were qualified to receive a full-mouth periodontal exam, which was conducted by certified dentists using a dental mirror and a Hu-Friedy periodontal probe. This exam provided information on periodontal pockets, recession, and loss of attachment.

According to Eke’s research [23], periodontitis can be classified as mild, moderate, or severe based on the amount of attachment loss (AL) and pocket depth (PD) at four interproximal sites per tooth. Mild periodontitis is defined as having at least two interproximal sites with an AL of 3 mm or more, and at least two interproximal sites with a PD of 4 mm or more (not on the same tooth) or one site with a PD of 5 mm or more. Moderate periodontitis is defined as having at least two interproximal sites with an AL of 4 mm or more (not on the same tooth) or at least two interproximal sites with a PD of 5 mm or more (not on the same tooth). Severe periodontitis is defined as having at least two interproximal sites with an AL of 6 mm or more (not on the same tooth) and at least one interproximal site with a PD of 5 mm or more. Failure to meet any of these criteria results in a diagnosis of no periodontitis.

### Albumin and covariates

Samples for serum albumin concentration analysis are acquired through venipuncture and must meet the following criteria: (1) Bilirubin levels should be below 30.0 mg/dL or lipemia below 3+. (2) Hemolysis levels should not exceed 4+. (3) Samples with lipemia levels above 3 + must undergo treatment with the Lipoclear Clarifying agent before analysis. Serum or plasma should not be stored at temperatures between + 15 °C and + 30 °C for more than 8 h. If assays cannot be completed within this timeframe, samples should be refrigerated at + 2 °C to + 8 °C. For storage beyond 48 h, or if assays are not completed within this period, samples must be frozen at −15 °C to −20 °C. Thawing frozen samples only once is crucial to prevent analyte deterioration from repeated freeze-thaw cycles. Albumin detection was performed using the Beckman Coulter UniCel® DxC800 Synchron Clinical System, employing a bichromatic digital endpoint method. This method involves the reaction of albumin with Bromcresol Purple reagent, forming a complex. Quantification is based on measuring absorbance change at 600 nm, providing a direct reflection of albumin concentration in the sample.

Covariates were selected based on their association with sNfL or periodontitis [24–29]. These included sociodemographic characteristics such as age (< 60 years, ≥ 60 years), sex (male, female), race (Non-Hispanic White, other races), marital status (married and living with a partner, single), education level (high school or above, less than high school), and PIR (≤ 1, 1–3, > 3). Additionally, lifestyle variables such as smoking status (never, former, current), drinking status (no, yes), sleep duration, BMI (< 25 kg/m^2^, ≥ 25 kg/m^2^), work activity (no, yes), and recreational activity (no, yes) were taken into consideration. Laboratory indices such as the estimated glomerular filtration rate (eGFR) and the SII were also included. The Charlson Comorbidity Index (CCI) was used to account for potential effects of disease status. It was calculated by summing up the scores of various diseases [30]. To determine BMI, the weight in kilograms was divided by the height in meters squared, and the eGFR was determined using the Chronic Kidney Disease Epidemiology Collaboration 2009 creatinine-based formula. The SII level was calculated by multiplying the platelet count by the neutrophil count and dividing it by the lymphocyte count [31].

### Statistical analysis

Survey weights were used in the analysis to account for the complex survey design of NHANES. Given the skewed distribution of sNfL, a logarithmic transformation was conducted to improve normality. The transformed data was then used for subsequent analysis. Continuous variables conforming to a normal distribution were presented as weighted means with standard errors (SE), while those not following a normal distribution were shown as weighted medians and interquartile ranges (IQR). Categorical variables were repre-sented as weighted proportions. The characteristics of the participants were evaluated through t-tests for normally distributed continuous variables, the Wilcoxon test for comparing non-normally distributed continuous variables, and chi-square tests for categorical variables. Multiple weighted linear regression models were used to explore the connection between sNfL and periodontitis. Model 1 was unadjusted, while model 2 was adjusted for age, sex, and race. Model 3 included the variables from model 2, as well as additional confounders such as marital status, educational level, PIR, BMI, sleep duration, SII, drinking status, smoking status, work activity, recreational activity, eGFR, and CCI. The RCS models were used to assess the dose-response relationship between sNfL and periodontitis. This included three knots located at the 10th, 50th, and 90th percentiles of sNfL levels, with the median sNfL level as the reference point. Furthermore, a stratified analysis was conducted to assess the stability of the results. Additionally, mediation analyses were performed to investigate the role of SA in the association between sNfL and periodontitis. The total effect (TE) of sNfL on periodontitis was divided into the direct effect (DE) and the indirect effect (IE). TE represents the overall impact of sNfL on periodontitis, while IE refers to the effect of sNfL on periodontitis through SA. DE, on the other hand, measures the effect of sNfL on periodontitis when controlling for SA. The proportion of IE in TE was calculated to determine the extent of mediation by SA.

Data analysis was conducted using R version 4.1.3, and the results were considered statistically significant if the p-value was less than 0.05.

## Results

### Characteristics of the study population

In the study, 1218 participants from the NHANES 2013–2014 were included, of whom 50.28% were male. Most of the participants were non-Hispanic White and under the age of 60. Table 1 shows significant differences (p-value < 0.05) in demographic and health-related factors between the non-periodontitis and periodontitis groups, including age, sex, race, marital status, educational level, PIR, smoking status, work activity, recreational activity, CCI, SA, and sNfL. Notably, individuals with periodontitis exhibited significantly higher CCI and sNfL levels compared to those without periodontitis (p-value < 0.05).

**Table 1:** General characteristics of participants by periodontitis status

Characteristics	Total	Non-periodontitis	Periodontitis	*p*-value
Age (%)				0.02
< 60 years	77.03	79.42	71.91	
≥ 60 years	22.97	20.58	28.09	
Sex (%)				0.001
Male	50.28	46.56	58.24	
Female	49.72	53.44	41.76	
Race (%)				0.002
Other race	31.64	25.59	44.61	
Non-Hispanic White	68.36	74.41	55.39	
Marital status (%)				< 0.001
Married and living with partner	70.99	74.53	63.38	
Single	29.01	25.47	36.62	
Educational level (%)				< 0.0001
High school or above	86.77	92.73	73.99	
Less than high school	13.23	7.27	26.01	
Drinking status (%)				0.72
No	9.98	9.69	10.61	
Yes	90.02	90.31	89.39	
PIR (%)				< 0.0001
≤ 1	14.48	9.47	25.22	
1–3	31.84	27.32	41.52	
> 3	53.68	63.21	33.26	
BMI (%)				0.85
< 25 kg/m^2^	23.67	23.90	23.20	
≥ 25 kg/m^2^	76.33	76.10	76.80	
Smoking status (%)				0.004
Never	57.28	63.40	44.15	
Former	25.19	23.74	28.31	
Now	17.53	12.80	27.54	
Work activity (%)				0.02
No	57.55	60.28	51.69	
Yes	42.45	39.72	48.31	
Recreational activity (%)				< 0.001
No	48.80	44.70	57.59	
Yes	51.20	55.30	42.41	
SII^*^(1000 cells/µL)	502.32 (10.01)	489.41 (14.32)	530.00 (13.84)	0.09
Sleeping duration^*^(h)	6.88 (0.04)	6.89 (0.05)	6.85 (0.11)	0.76
CCI^*^	1.05 (0.05)	0.95 (0.04)	1.25 (0.11)	0.02
eGFR^*^(mL/min/1.73m^2^)	92.23 (0.86)	91.90 (1.09)	92.92 (0.96)	0.46
SA^*^(g/L)	42.45 (0.17)	42.73 (0.18)	41.86 (0.22)	0.003
sNfL^**^(pg/mL)	1.10 (0.95,1.29)	1.08 (0.94,1.25)	1.16 (1.00,1.38)	< 0.001

*Abbreviations* sNfL, Serum neurofilament light chain; PIR, Income-to-poverty ratio; BMI, Body mass index; CCI, Charlson comorbidity index; SA, Serum albumin; eGFR, Estimated glomerular filtration rate; SII, Systemic immunity-inflammation index

Categorical variables are represented as proportions. Normally distributed continuous variables are shown as means (SE), and non-normally distributed continuous variables are displayed as weighted medians and interquartile ranges (IQR). sNfL was log10-transformed.

The p-values with ^*^ were calculated using the t-test, while the p-values with ^**^ were determined using the Wilcoxon test. The remaining p-values were based on the χ^2^ test.

### Association between sNfL and periodontitis

**Fig. 1:**
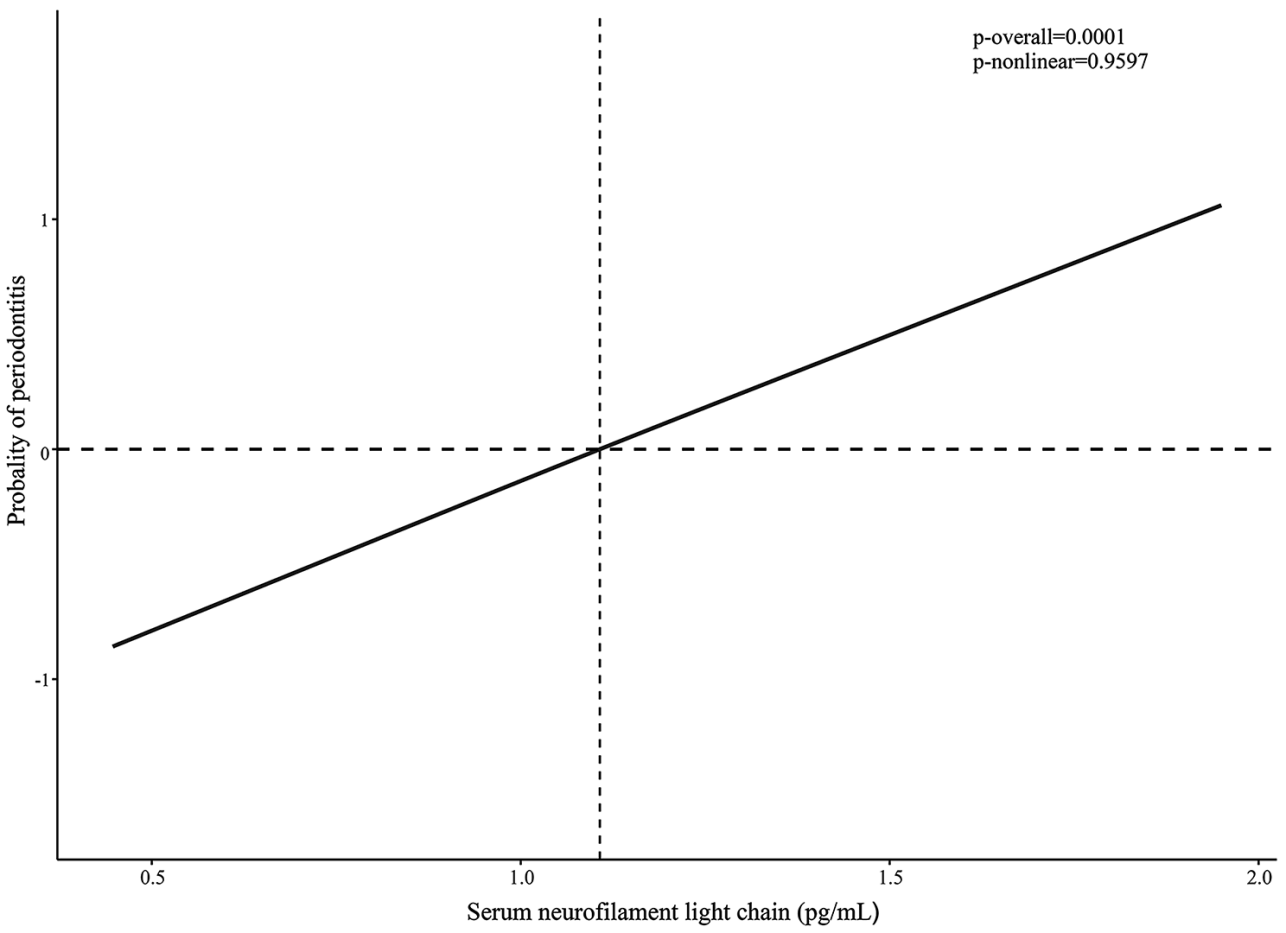
Analysis of Restricted Cubic Spline Regression

Three models were developed to assess the relationship between sNfL levels and periodontitis in this study (Table 2). The results consistently indicated a positive association between sNfL levels and periodontitis across all three models: model 1 (OR, 3.08; 95% CI, 1.48 to 6.39, p-value = 0.01), model 2 (OR, 3.69; 95% CI, 1.73 to 7.90, p-value = 0.003), and model 3 (OR, 3.58; 95% CI, 1.52 to 8.43, p-value = 0.01). Furthermore, the RCS models revealed a linear correlation between sNfL and periodontitis (Fig. 1).

**Table 2:** Association between sNfL and periodontitis

	Model 1		Model 2		Model 3	
	OR, 95%CI	p-value	OR, 95%CI	p-value	OR, 95%CI	p-value
sNfL(pg/mL)	3.08(1.48,6.39)	0.01	3.69(1.73,7.90)	0.003	3.58(1.52,8.43)	0.01

*Abbreviations* sNfL, serum neurofilament light chain; OR, odds ratio; CI, confidence interval

sNfL was log10-transformed

Model 1: No variables were adjusted

Model 2: Adjusted for age, sex, race

Model 3: Adjusted for age, sex, race, marital status, educational level, PIR, BMI, sleep duration, drinking status, smoking status, work activity, recreational activity, SII, eGFR and CCI

### Associations of SA with sNfL and periodontitis

Table 3 displays the associations of SA with sNfL and periodontitis. The results demonstrate a significant and inverse correlation between sNfL and SA in all three models (p-value < 0.05). The results also suggest that a 1-unit increase in SA is associated with an 8% decrease in the relative risk of developing periodontitis after adjusting for all covariates (p-value < 0.05).

**Table 3:** Associations of SA with sNfL and periodontitis

	Model 1		Model 2		Model 3	
	OR, 95%CI	p-value	OR, 95%CI	p-value	OR, 95%CI	p-value
sNfL	−2.12(−3.26, −0.98)	0.001	−2.40(−3.62, −1.18)	0.001	−2.09(−3.48, −0.70)	0.01
Periodontitis	0.91(0.86, 0.96)	0.004	0.89(0.82, 0.96)	0.005	0.92(0.86, 0.98)	0.01

*Abbreviations* sNfL, serum neurofilament light chain; OR, odds ratio; CI, confidence interval

sNfL was log10-transformed

Model 1: No variables were adjusted

Model 2: Adjusted for age, sex, race

Model 3: Adjusted for age, sex, race, marital status, educational level, sleep duration, PIR, BMI, drinking status, smoking status, work activity, recreational activity, SII, eGFR and CCI.

### Stratified analysis

**Fig. 2:**
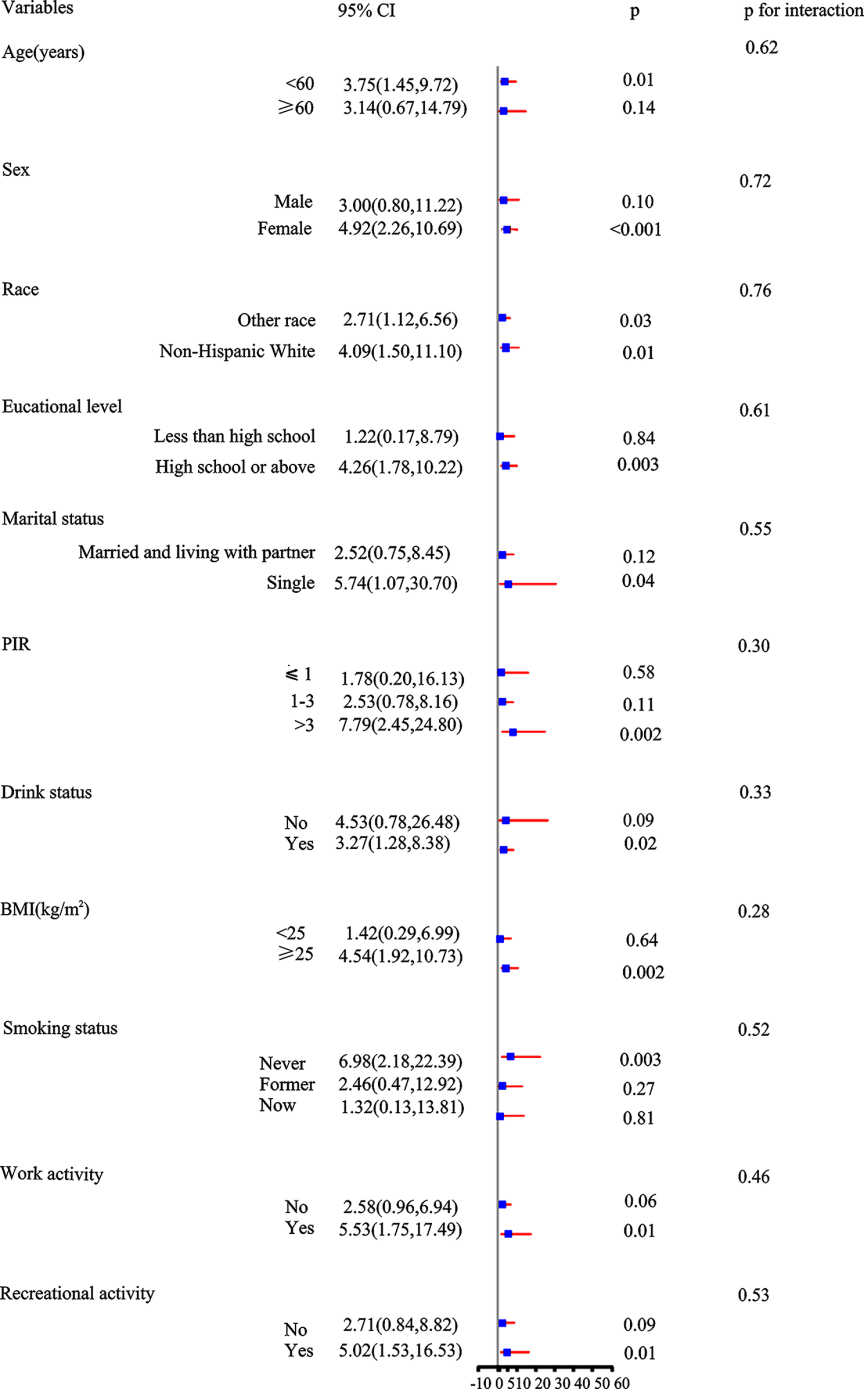
Stratified analysis of the association between sNfL and risk of periodontitis

The results of the stratified analysis, illustrated in Fig. 2, suggested that sNfL is a risk factor for periodontitis, even when all covariates were taken into consideration. Furthermore, the association was not affected by variables such as age, sex, race, marital status, educational level, PIR, BMI, drinking status, smoking status, work activity, or recreational activity (all p-values for interaction > 0.05)

### Mediation analysis

**Fig. 3:**
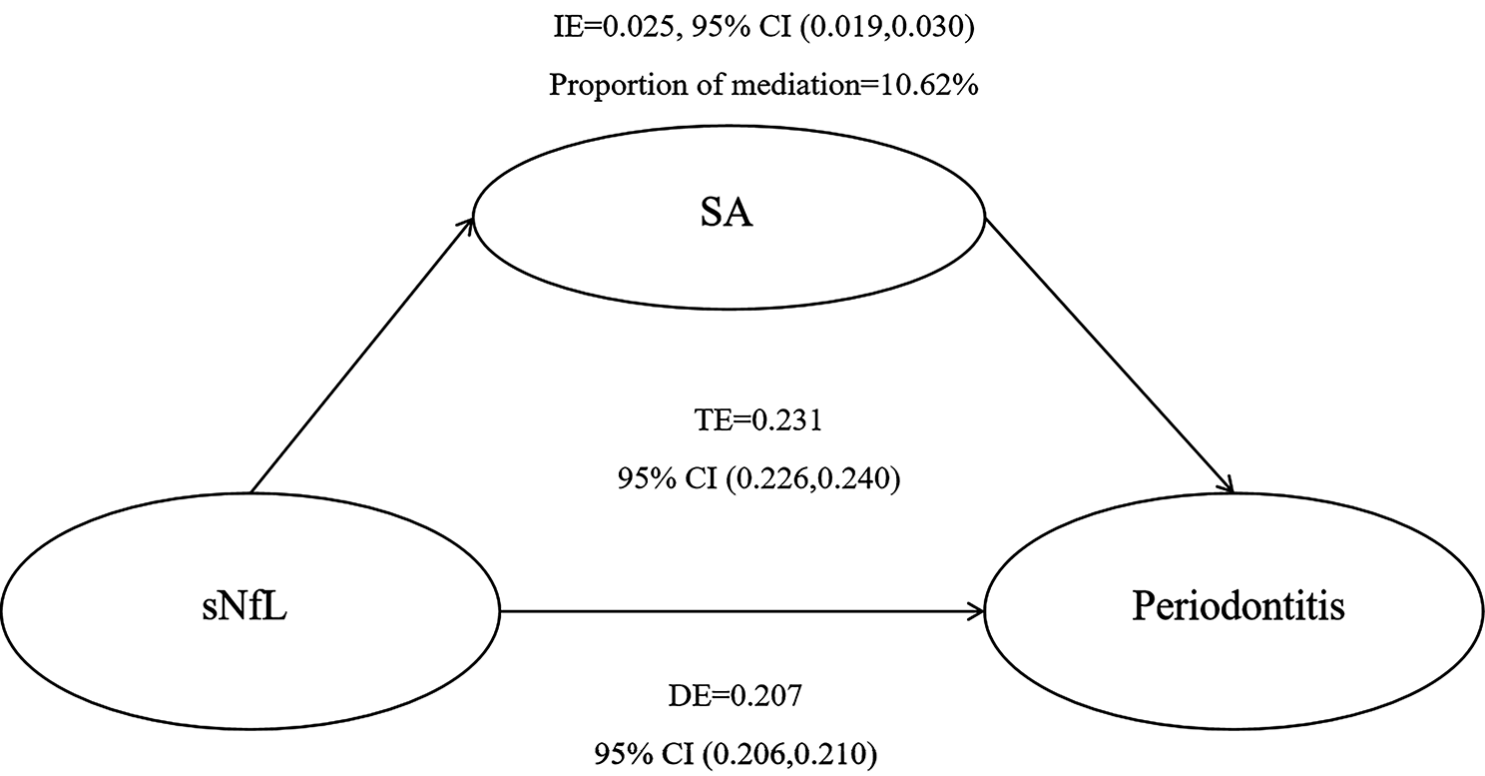
Mediation analysis of serum albumin on the association between sNfL and periodontitis

The mediators in the relationship between sNfL and periodontitis were identified as SA, with a mediation proportion of 10.62% (Fig. 3)

## Discussion

In this investigation, we established a significant positive correlation between sNfL and periodontitis within a nationally representative US population. After controlling for potential confounders, the findings remained consistent across all models. Moreover, the RCS model showed a linear relationship. Stratified analysis of the link between sNfL and periodontitis revealed consistent results across all subgroups, with no evidence of interaction. Additionally, SA was found to mediate the effect of sNfL on periodontitis, with a mediation proportion of 10.62%

Epidemiologically, periodontitis, characterized by significant inflammation of the oral mucosa, has been linked to various conditions, particularly NDs. A systematic review demonstrated a six-fold increased risk of cognitive decline in individuals with periodontitis [32]. Research conducted in Geneva revealed signs of local inflammation in the oral cavities of Parkinson’s disease patients, despite good dental and periodontal health [33]. A study involving 316 multiple sclerosis patients and 1580 controls found that females with multiple sclerosis had a higher risk of chronic periodontitis compared to female controls [34]. Furthermore, a study of 109 participants established a significant association between periodontal disease and epilepsy, with individuals with periodontal disease experiencing more severe seizures [35]. Our study identified a linear correlation between sNfL levels and periodontitis, partially corroborating previous evidence while also suggesting the possibility of sNfL as a biomarker in non-neurological diseases

The relationship between NDs and periodontitis is subject to ongoing debate, with a potential bidirectional association between them. In the case of Alzheimer’s disease (AD), studies have indicated that individuals with AD often exhibit poorer dental health compared to elderly individuals without the condition [36, 37], likely due to challenges in self-care as dementia progresses, along with potential links to systemic inflammation [38]. Conversely, research has shown that patients with dementia tend to have significantly worse clinical periodontal outcomes than those without systemic health issues [39]. Proposed mechanisms linking periodontitis to dementia include bacterial migration from the periodontal region to the bloodstream and other organs, as well as the potential harm caused by microbial toxins and inflammatory mediators to the vascular system [39, 40]. Furthermore, keratin could potentially be a contributing factor. NfL represents a type of intermediate filament and serves as a marker in NDs [41]. Oral epithelial cells rely on a stable configuration of 10-nm intermediate filaments for survival and proliferation, with keratin and the extracellular matrix providing support to maintain cellular structure. It has been observed that the degradation of cytokeratin-6 by lysine-specific proteases leads to the collapse of the cytoskeleton of rat gingival epithelial cells, resulting in cellular changes and inflammation similar to those of periodontitis tissue [42]. Besides the factors discussed above, oxidative stress also contributes to the onset of periodontitis [43]. Bone loss has been linked to oxygen free radicals, which can lead to lipid peroxidation during phagocytosis and damage proteins and DNA. This can cause inflammation, osteoporosis, and detachment of periodontal tissue. Additionally, the generation of reactive oxygen species has been associated with heightened expression of pro-inflammatory cytokines, which play a role in connective tissue damage and bone resorption [44]. However, an alternative perspective posits no association between periodontitis and AD, supported by two genome-wide association studies [45, 46] and the absence of a correlation between the APOE4 allele (a recognized risk factor for AD) and periodontitis [47]. The disparities observed may be partially attributed to variations in study design, highlighting the pressing need for more rigorous investigations to elucidate the relationship between periodontitis and sNfL or NDs.

The research revealed a significant inverse relationship between SA and both sNfL and periodontitis in individuals aged 30 and over. This finding confirms the idea that pathological conditions often display lower levels of SA. A large cohort study conducted in Switzerland showed that low SA levels independently predicted a 30-day mortality rate [48]. Results from a population-based study showed that individuals with high levels of SA concentration had a significantly lower risk of depression after adjusting for various variables [49]. A cohort study determined that albumin levels were significantly related to worse clinical outcomes when diagnosing amyotrophic lateral sclerosis. Additionally, albumin levels correlated with measurements of inflammation [50]. The association between SA and periodontitis has been established. Maruyama et al. found a strong association between SA concentration and the average clinical attachment levels in individuals with head and neck cancer [15]. Yoshihara et al. discovered a link between root caries and SA concentration in older adults [51]. Ogawa et al. suggested that there may be a reverse relationship between periodontal disease and SA concentration in elderly subjects [16]. The results showed that SA mediated the effect of sNfL on the risk of periodontitis, with a mediation proportion of 10.62%. This is consistent with the reported statement, potentially due to its functions as a carrier, chaperone, antioxidant, amino acid source, and osmoregulator, establishing it as a significant contributor to overall health status [11]

The study has several advantages. First, it is the first study to explore the relationship between sNfL, SA, and periodontitis simultaneously in a nationally representative sample. Second, we adjusted for potential confounders simultaneously to avoid bias. Third, SA was found to play a mediating role in the correlation between sNfL and periodontitis, potentially providing new insights into the underlying biological mechanisms. However, the study also has limitations. First, the current investigation is a cross-sectional study, which does not allow for determining the causal relationship between sNfL and periodontitis. Therefore, to confirm the findings, a subsequent large-scale cohort study is required. Second, the study encompassed individuals above the age of 30, and further study is required to determine the generalizability of the findings to younger populations

## Conclusions

sNfL levels are associated with periodontitis in individuals. This finding indicates that sNfL may be a predictor of periodontitis development. Furthermore, implementing nutritional interventions such as SA interventions could potentially mitigate the incidence of this condition. Further prospective studies are required to confirm and elucidate the underlying biological mechanisms

## Data Availability

The information from this research can be accessed through the free NHANES database, located at https://www.cdc.gov/nchs/nhanes/default.aspx.
